# The sequence of 12 Gy total body irradiation and cyclophosphamide does not impact outcomes in AML patients receiving myeloablative allogeneic stem cell transplantation

**DOI:** 10.1038/s41409-025-02755-3

**Published:** 2025-12-02

**Authors:** Georg-Nikolaus Franke, Jule Ussmann, Donata Backhaus, Nils Henrik Nicolay, Thomas Kuhnt, Franziska Nägler, Jacob Jendro, Vladan Vučinić, Marco Herling, Birthe Schetschorke, Carmen Herling, Saskia Weibl, Uwe Platzbecker, Klaus H. Metzeler, Sebastian Schwind, Madlen Jentzsch

**Affiliations:** 1 University of Leipzig Medical Center, Department for Hematology, Cellular Therapies and Hemostaseology, Leipzig, Germany; 2 University of Leipzig Medical Center, Department for Radiation Therapy, Leipzig, Germany; 3 University Hospital of Dresden, Dresden, Germany

**Keywords:** Acute myeloid leukaemia, Prognosis

**To the Editor:**

Allogeneic hematopoietic stem cell transplantation (HSCT) is considered standard of care in eligible patients with acute myeloid leukemia (AML) and intermediate or adverse risk in first complete remission (CR) as well as in patients suffering relapse according to the most recent European LeukemiaNet (ELN) 2022 recommendations [1]. The optimal preparative regimen in patients eligible for myeloablative conditioning (MAC) is still a matter of debate. Major concerns about using total body irradiation (TBI) are its potential long-term toxicities, including neurocognitive impairment, treatment-related cancers, as well as secondary endocrine, renal, and pulmonary organ damages [2–4]. Still, high dosage of TBI facilitates engraftment, provides a different mode of anti-cancer activity against chemotherapy-resistant clones, and includes sites poorly reached by chemotherapy as the central nervous system and testes. A high heterogeneity of MAC TBI techniques have been shown by the EBMT between centers, complicating the interpretation of trials incorporating TBI [5]. Cyclophosphamide 60 mg/kg bodyweight (BW) on two consecutive days followed by 12 Gy TBI, fractionated in six sessions over three days, is one of the most often used MAC regimen [6–10]. Due to unavailability of TBI on public holidays and weekends as well as limited apheresis slot availability influencing the transplant date, the order of cyclophosphamide and TBI frequently has to be exchanged to avoid postponing the transplant date with subsequent risks to the recipient. However, this may lead to distinct rates of complications, as mucosal damage, sinusoidal obstruction syndrome (SOS) and infections, potentially causing a higher non-relapse mortality (NRM). A previous Chinese analysis in 88 patients with lymphoma, acute lymphoblastic leukemia or chronic myeloid leukemia showed similar overall survival (OS), but suggested lower short-term radiation toxicity and higher compliance in patients receiving TBI before chemotherapy. However, this analysis included a highly heterogenous group of patients treated with autologous and allogeneic HSCT, TBI doses between 7-11 Gy and various chemotherapies, and did not comprise AML patients [11]. Aiming to fill this gap of knowledge, we retrospectively analyzed 144 consecutive AML patients receiving an allogeneic MAC HSCT at our center between 2001 and 2018. All AML patients were homogeneously treated with 12 Gy TBI and 120 mg/kg body weight cyclophosphamide. As TBI was unavailable on weekends at our center, the chemotherapy/TBI sequence depended on donor and apheresis slot availability: 90 patients (63%) received cyclophosphamide 60 mg/kg BW on days -5 and -4 followed by fractionated TBI (2 × 2 Gy per day) from day -3 to -1 (cyclophosphamide-TBI group), while 56 patients (37%) received TBI from day -6 to -4 followed by cyclophosphamide on days -3 and -2 (TBI-cyclophosphamide group, Fig. 1a) before HSCT. Median age at HSCT was 43 (range 17–58) years. The majority of patients (81%) had de novo AML and were transplanted in first composite CR (CRc, *i.e*. CR with or without hematologic recovery, 79%), 18% were in later CRc and 3% were transplanted with active disease. Donors were 8/8 HLA-matched siblings (MSD, 38%) or matched (MUD, 54%) or mismatched unrelated (MMUD, 8%) donors. All patients received peripheral blood stem cells. Immunosuppression and antimicrobial prophylaxis did not differ between both treatment groups (for details see Supplementary Information). Full patient and HSCT characteristics are shown in Supplementary Table S1. Median follow up after HSCT was 4.5 years. Genetic analyses at diagnosis, measurable residual disease (MRD) evaluation at HSCT, and statistical analyses are described in the Supplementary Information.

**Fig. 1:**
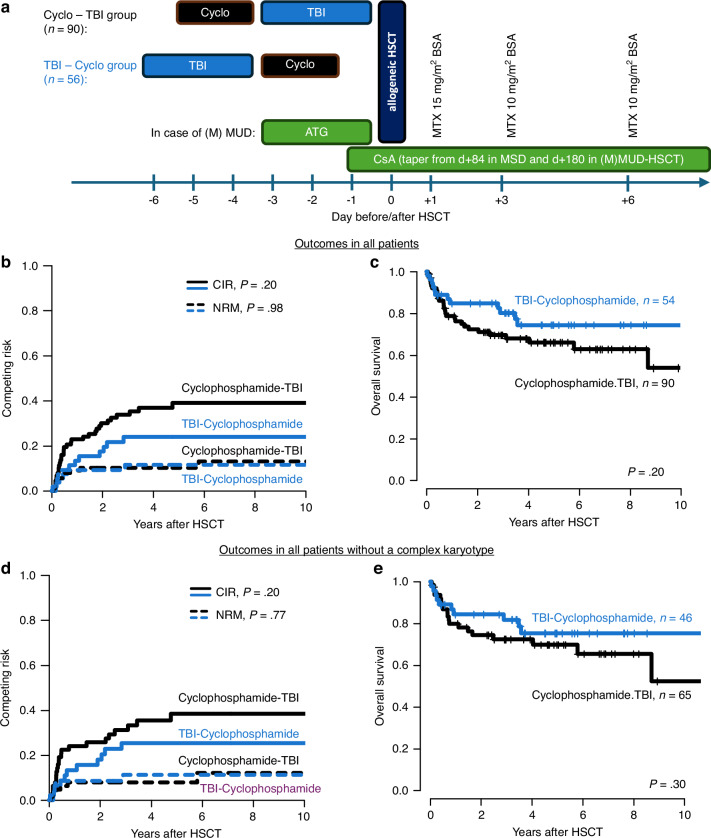
Scheme of conditioning and immunosuppression and outcomes according to the sequence of cyclophosphamide and TBI. **a** Schema of conditioning and immunosuppression. **b** Cumulative incidence of relapse (CIR) and non-relapse mortality (NRM), and **c** Overall survival (OS) **in all patients**. **d** CIR and NRM and **e** OS **in patients without a complex karyotype**. ATG anti thymocyte globulin (Grafalon ®), BSA body surface area, Cyclo cyclophosphamide, CsA cyclosporine A, HSCT hematopoietic stem cell transplantation, MSD matches sibling donor, MTX methotrexate, (M)MUD (mis) matched unrelated donor, TBI total body irradiation.

For the whole cohort, 5-year OS, cumulative incidence of relapse (CIR), NRM, and OS was 33%, 11%, and 69%, respectively. The sequence of 12 Gy TBI and cyclophosphamide did not impact NRM (*P* = 0.98) or OS (*P* = 0.20, Fig. 1b, c). Although we observed a trend for a higher CIR in the cyclophosphamide-TBI group (*P* = 0.09), this group also had a higher incidence of complex karyotypes (*P* = 0.02, Supplementary Table S1), and likely a higher baseline relapse risk. After adjusting for these differences in a multivariate analysis (Supplementary Table S2) as well as in a subgroup analysis of patients without a complex karyotype (Fig. 1c, d), CIR, NRM, and OS did not differ with regard to the sequence of TBI and cyclophosphamide. Also the incidence of acute graft-versus-host disease (GvHD, *P* = 0.50) or chronic GvHD (*P* = 0.84) was similar between patients receiving cyclophosphamide-TBI or TBI-cyclophosphamide (Supplementary Table S1). The only notable difference was a significantly longer time to platelet (median 14 *vs.* 13 days, *P* = 0.02), but not leukocyte engraftment (*P* = 0.48, Supplementary Fig. S1C, D) in the cyclophosphamide-TBI group in patients receiving a graft from an unrelated donor. Still, no distinct engraftment times for leukocytes or platelets were observed in the whole cohort (*P* = 0.62 and *P* = 0.25, respectively, Supplementary Fig. S1A, B) or in patients transplanted from an MSD (Supplementary Fig. S2).

Also in relevant subgroup analyses within the three ELN 2022 risk groups (Supplementary Fig. S3), in patients transplanted in first or later CRc (Supplementary Fig. S4), or in MRD-positive or negative patients at HSCT (Supplementary Fig. S5) the sequence of TBI and cyclophosphamide did not associate with CIR, NRM or OS.

In contrast, known risk factors for outcomes in AML patients undergoing allogeneic HSCT, such as the ELN 2022 risk group, disease origin or the morphologic remission and MRD status at the time of HSCT retained their prognostic impact for the whole cohort (Supplementary Table S2 and Supplementary Fig. S5). In our analysis, also patients aged over 40 years at the time of allogeneic HSCT had shorter OS due to a higher NRM than younger individuals in univariate (Supplementary Fig. S6A, B) and multivariate analyses. Bornhäuser et al. presented a study randomizing 99 patients younger than 60 years with AML in first CR between a conditioning regimen with 12 Gy TBI/cyclophosphamide and 8 Gy TBI/ fludarabine [12]. Here, patients receiving 12 Gy TBI/cyclophosphamide had a lower CIR but higher NRM, resulting in a similar OS rate for both groups. In this trial, subgroup analyses according to age under or over 40 years at HSCT showed the higher NRM to be restricted to patients older than 40 years at HSCT, further strengthening the use of less intensive regimens in these patients. When we analyzed subgroups of patients younger or older than 40 years at HSCT - after excluding patients with a complex karyotype due to their uneven distribution between groups - again, the sequence of TBI and cyclophosphamide did not significantly impact outcomes (Supplementary Fig. 6C, D).

Limitations of our analysis are its retrospective nature with a consecutive lack of data on short-term mucosal damage, organ toxicities, or SOS and long-term TBI-related events as well as restricted number of patients, especially in subgroup analyses. However, as TBI techniques can vary significantly between centers, a monocentric analysis may also have the advantage of excluding further bias derived from distinct modes of TBI application. In conclusion, despite the mentioned limitations, our study provides evidence that the sequence of TBI and cyclophosphamide does not impact long-term outcomes after allogeneic HSCT in AML patients. Subsequently, it seems safe to interchange TBI and cyclophosphamide as needed to allow the planning of allogeneic MAC HSCT with 12 Gy TBI at the earliest time point at which a stem cell donation can be provided by the apheresis center.

## Supplementary information

Supplemental Material

## Data Availability

Data presented in this study may be available upon request from the corresponding author. The study was conducted according to the guidelines of the Declaration of Helsinki. Data analyses were approved by the Institutional Review Board of the University Hospital Leipziig (536/197 on 03-03-1997, 629/1997 on 10-13-1997, 027/2002 on 02-11-2002) and amended on 02-01-2008, 162/2004 on 05-04-2005 and amended on 11-25-2010, and 363/16-ek on 11-07-2016.

## References

[1] Döhner H, Wei AH, Appelbaum FR, Craddock C, DiNardo CD, Dombret H, et al. Diagnosis and management of AML in adults: 2022 recommendations from an international expert panel on behalf of the ELN. Blood. 2022;140:1345–77. 35797463 10.1182/blood.2022016867

[2] Kwon J, Kim BH. Long-term toxicities after allogeneic hematopoietic stem cell transplantation with or without total body irradiation: a population-based study in Korea. Radiat Oncol J. 2024;42:50–62. 38549384 10.3857/roj.2023.00871 PMC10982063

[3] Gruen A, Exner S, Kühl J-S, Von Stackelberg A, Budach V, Stromberger C et al. Total body irradiation as part of conditioning regimens in childhood leukemia-long-term outcome, toxicity, and secondary malignancies of the blood. 10.1007/s00066-021-01810-4. 10.1007/s00066-021-01810-4 PMC8760188 34282476

[4] Sieker K, Fleischmann M, Trommel M, Ramm U, Licher J, Bug G, et al. Twenty years of experience of a tertiary cancer center in total body irradiation with focus on oncological outcome and secondary malignancies. Strahlentherapie und Onkol. 2022;198:547–57. 10.1007/s00066-022-01914-5 PMC9165288 35318487

[5] Giebel S, Miszczyk L, Slosarek K, Moukhtari L, Ciceri F, Esteve J, et al. Extreme heterogeneity of myeloablative total body irradiation techniques in clinical practice: A survey of the Acute Leukemia Working Party of the European Group for Blood and Marrow Transplantation. Cancer. 2014;120:2760–5. 24804873 10.1002/cncr.28768

[6] Riddell S, Appelbaum FR, Buckner CD, Stewart P, Clift R, Sanders J, et al. High-dose cytarabine and total body irradiation with or without cyclophosphamide as a preparative regimen for marrow transplantation for acute leukemia. J Clin Oncol. 1988;6:576–82. 3282031 10.1200/JCO.1988.6.4.576

[7] Luo C, Wu G, Huang X, Ding Y, Huang Y, Song Q, et al. Myeloablative conditioning regimens in adult patients with acute myeloid leukemia undergoing allogeneic hematopoietic stem cell transplantation in complete remission: a systematic review and network meta-analysis. Bone Marrow Transpl. 2023;58:175–85. 10.1038/s41409-022-01865-6 36357773

[8] Gupta T, Kannan S, Dantkale V, Laskar S. Cyclophosphamide plus total body irradiation compared with busulfan plus cyclophosphamide as a conditioning regimen prior to hematopoietic stem cell transplantation in patients with leukemia. Hematol Oncol Stem Cell Ther. 2011;4:17–29. 21460603 10.5144/1658-3876.2011.17

[9] Thomas ED, Buckner CD, Banaji M, Clift RA, Fefer A, Flournoy N, et al. One hundred patients with acute leukemia treated by chemotherapy, total body irradiation, and allogeneic marrow transplantation. Blood. 1977;49:511–33. 14751

[10] Thomas ED, Buckner CD, Clift RA, Fefer A, Johnson FL, Neiman PE, et al. Marrow transplantation for acute nonlymphoblastic leukemia in first remission. N Engl J Med. 1979;301:597–9. 381925 10.1056/NEJM197909133011109

[11] Li D-Z, Kong P-Y, Sun J-G, Wang X-X, Li G-H, Zhou Y-B, et al. Comparison of Total Body Irradiation Before and After Chemotherapy in Pretreatment for Hematopoietic Stem Cell Transplantation. Cancer Biother Radiopharm. 2012;27:119–23. 22149642 10.1089/cbr.2011.1041 PMC3304241

[12] Bornhäuser M, Kienast J, Trenschel R, Burchert A, Hegenbart U, Stadler M, et al. Reduced-intensity conditioning versus standard conditioning before allogeneic haemopoietic cell transplantation in patients with acute myeloid leukaemia in first complete remission: a prospective, open-label randomised phase 3 trial. Lancet Oncol. 2012;13:1035–44. 22959335 10.1016/S1470-2045(12)70349-2

