# Supplementary material for: The sequence of 12 Gy total body irradiation and cyclophosphamide does not impact outcomes in AML patients receiving myeloablative allogeneic stem cell transplantation
Source: Bone Marrow Transplant. 2025 Dec 2;61(2):235–7. doi: 10.1038/s41409-025-02755-3 (PMC12909114; doi:10.1038/s41409-025-02755-3)
Supplement: Supplementary file 1 — Supplemental Material [file 41409_2025_2755_MOESM1_ESM.docx]

Supplementary Information to

**The sequence of 12 Gy total body irradiation and cyclophosphamide does not impact outcomes in AML patients receiving myeloablative allogeneic stem cell transplantation**

Franke *et al*.

**Patients and methods**

Treatment protocols

The majority of patients with acute myeloid leukemia (AML) received standard Cytarabine-based induction protocols as first line treatment. These were conventional 7+3 (n= 33), or treatment within or according to the OSHO studies (061 or #069, under or over 60 years, n= 105),^1,2^ the Ratify Trial (n= 5)^3^, or the Quantum first trial (n=1).^4^

Cytogenetic and Molecular Analyses

Cytogenetic analyses at diagnosis were performed using standard techniques of banding and *in situ* hybridization (FISH). The mutation status of the genes *NPM1*, *FLT3*-ITD and -TKD was determined as described before.^5^ In patients with stored DNA samples available, the diagnostic mutation status of 54 genes recurrently mutated in myeloid malignancies was evaluated using next generation sequencing (Illumina, San Diego, CA, USA) as previously described.^6^ Patients were grouped according to the European LeukemiaNET (ELN) 2022 recommendations into favorable (34%), intermediate (31%), and adverse (35%) risk at diagnosis.^7^

In 73 patients stored remission samples were available to evaluate the measurable residual disease (MRD) status up to 28 days prior to allogeneic hematopoietic stem cell transplantation (HSCT). Molecular MRD was assessed using digital polymerase chain reaction (PCR), for at least one of the targets *NPM1* mutation, *BAALC/ABL1* copy numbers and *MN1/ABL1* copy numbers or using quantitative reverse transcriptase PCR for *WT1* expression levels adapting the previously published cut-offs.^8–11^ Molekular MRD positivity was defined as being positive for any analyzed marker. In patients without material for molecular MRD analysis, FISH of at least 100 interphases of the dominant cytogenetic aberration at diagnosis was used as cytogenetic MRD at HSCT as previously published.^12^

Antimicrobial prophylaxes and supportive therapy

Patients were treated in a high-efficiency air-filtered room. Central-venous catheters were placed before the start of the conditioning regimen.

All patients received trimethoprim-sulfamethoxazole for *Pneumocystis jirovecii* prophylaxis prior to conditioning chemotherapy and after complete hematologic regeneration. Additionally, patients received ciprofloxacin for antibacterial prophylaxis, valacyclovir for antiviral prophylaxis and fluconazole for antifungal prophylaxis from the start of the conditioning regimen. Red blood cell and platelet transfusions were administered preemptively to maintain hemoglobin levels greater to 7 g/dl and platelet counts greater to 15,000/µl. All blood products were filtered, irradiated and CMV negative for patients tested CMV negative prior to start of conditioning regimen.

Total Body Irradiation

Total body irradiation (TBI) was administered with twice daily fractions of 2 Gy to a total of 12 Gy using a dose rate of 500 MU/min from linear accelerators on three consecutive days. The lungs were attenuated in all patients to a dose of 10 Gy. The rate of TBI delivery remained the same through the whole study period.

Immunosuppression and graft-versus-host disease

For prevention of graft-versus-host disease (GvHD), all patients received an intravenous starting dose of 5 mg/kg body weight cyclosporine A in two daily doses from day -1 which was adjusted to a whole-blood target level of 200 ng/ml. Patients additionally received methotrexate 15 mg/m² body surface area intravenously on days +1, and 10 mg/m² body surface area intravenously on days +3 and +6 after HSCT. Patients with an unrelated matched (MUD) or mismatched (MMUD) donor additionally received *in vivo* T-cell depletion with 10-20 mg/kg body weight antithymocyte globuline (ATG, Grafalon) per day for three days. Detailed information is depicted in Figure 1A. Cyclosporine A was reduced starting on day +84 or day +180 following related or unrelated HSCT, respectively. Patients were evaluated for incidence of acute GvHD and chronic GvHD using established criteria of the Glucksberg grading system.^13^ Immunosuppression was prolonged or extended with systemic steroids in cases of GvHD (grade > 2 according to Glucksberg grading system). Requirement for acute GvHD was engraftment while requirement for chronic GvHD was engraftment and survival for at least 100 days after HSCT.

Definition of disease response

According to the ELN 2022 risk classification, complete remission (CR) was defined as the presence of <5% blasts in bone marrow, neutrophils >1.0 x 10^9^/L, platelets >100 x 10^9^/L, independence of blood transfusion and no extramedullary disease.^7^ CR with incomplete hematologic recovery (CRi) or partial hematologic recovery (CRh) was defined as CR with platelets <100 x 10^9^/L or neutrophils <1.0 x 10^9^/L and CR with platelets >50 x 10^9^/L and neutrophils >0.5 x 10^9^/L. Finally, morphologic leukemia-free state (MLFS) required no hematologic recovery. CR, CRi, CRh and MLFS were summarized as composite complete remission (CRc). In patients receiving allogeneic HSCT, the presence of CRc was confirmed within 28 days prior to HSCT by bone marrow and peripheral blood analysis. Active disease at HSCT was defined by a persisting blast count >5% in bone marrow, persisting blasts in peripheral blood or the detection of extramedullary disease.

Statistical analyses

Overall survival (OS) was calculated from HSCT until death from any cause using the Kaplan-Meier method and group comparisons using the log-rank test. Competing risk analyses for the cumulative incidence of relapse (CIR) and non-relapse mortality (NRM) were calculated from HSCT until relapse or death without relapse using Fine and Gray method.^14^ Associations with baseline clinical and genetic as well as with HSCT-related characteristics were compared using the Kruskal-Wallis-Test and Fisher’s exact test for continuous and categorical variables, respectively. All P-values are 2-sided and the type I error is 5%. Statistical analyses were performed using the R statistical software platform (version 4.3.3).^15^

Multivariate analyses

We constructed multivariable proportional hazard models for CIR, NRM, and OS to evaluate the impact of the used sequence of cyclophosphamide and TBI by backwards adjusting for other variables. In addition to the used sequence in conditioning, the following variables were considered for multivariable analyses: sex, disease origin (*de novo* *vs* secondary or treatment-related), ELN 2022 genetic risk, age at HSCT (<40 years *vs* ≥ 40 years), HCT-CI score (≥ 3 *vs* 1/2 *vs* 0), remission status at HSCT (CRc *vs* no CRc), donor type (related *vs* unrelated), HLA match (antigen match *vs* mismatch), CMV risk (recipient positive/donor negative *vs* all others) and donor sex (female to male *vs* all others). Of these, variables significant at α=.10 in univariable analyses were considered for multivariable analyses. Factors significant at 5% level were kept in the final model. Hazard ratios with their corresponding 95% confidence intervals (CI) were indicated for every significant prognostic factor.

**Results**

Patient characteristics according to conditioning sequence

Patients in the cyclophosphamide-TBI group were significantly more likely to have a complex karyotype (20% *vs* 4%, *P*=.02), whereas *FLT3-*TKD mutations were more common in the TBI-cyclophosphamide group (31% *vs* 11%, *P*=.02). There were no other significantly different clinical, genetic, or HSCT-related characteristics between both groups (Supplementary Table S1).

Engraftment and GvHD

All patients engrafted. Comparing the cyclophosphamide-TBI and TBI-cyclophosphamide groups within the whole patient cohort, neither the time to leukocyte engraftment (median 15 days *vs* 14 days, *P*=.62) nor the time to platelet engraftment (median 13 days *vs* 13 days, *P*=.25, Supplementary Figure S1A,B) differed significantly. This also remained true when patients transplanted from a matched sibling donor (MSD) were regarded separately (leukocyte engraftment: median 13.5 days *vs* 13.5 days, *P*=.60 and platelet engraftment: median 13 *vs* 13.5 days, *P*=.45, Supplementary Figure S2). However, in patients engrafted from an MUD or MMUD, the time to platelet engraftment was significantly longer when the sequence cyclophosphamide-TBI was used (median 14 days *vs* 13 days, *P*=.02, Supplementary Figure S1C), while, again, the time to leukocyte engraftment was similar (median 15 *vs* 14 days, *P*=.48, Supplementary Figure S1D).

With regards to GvHD, the incidence of acute GvHD (*P*=.50) as well as chronic GvHD (*P*=.84) did not differ between patients receiving cyclophosphamide-TBI or TBI-cyclophosphamide (Supplementary Table S1).

Causes of death

A total of 41 patients (28%) died, 29 (32%) after receiving the cyclophosphamide-TBI, and 12 (22%) after receiving the TBI-cyclophosphamide sequence. Twenty-one patients – 14 after receiving cyclophosphamide-TBI and 7 after TBI-cyclophosphamide - died after AML relapse or progression, 20 patients – 12 after cyclophosphamide-TBI and 8 after TBI-cyclophosphamide – died without relapse from GvHD and/or infection, and 4 patients – 3 after cyclophosphamide-TBI and 1 after TBI-cyclophosphamide – died from other causes (3 from cardiovascular events and 1 after developing a secondary malignancy).

Impact of age at HSCT

Patients 40 years or older at the time of HSCT had significantly higher non-relapse mortality in (*P*=0.01) and shorter OS (*P*=0.02, Supplementary Figure S6A,B) than younger patients. Also in subgroup analyses according to age - excluding patients with complex karyotype due to the uneven distribution between groups - the sequence of TBI and cyclophosphamide did not significantly impact patient outcomes, both in patients younger than 40 years (CIR *P*=.79, NRM *P*=.13, and OS *P*=.50, Supplementary Figure S6C) or older than 40 years at HSCT (CIR *P*=.13, NRM *P*=.82, and OS *P*=.10, Supplementary Figure S6D).

References:

1 Büchner T, Schlenk RF, Schaich M, Döhner K, Krahl R, Krauter J *et al.* Acute Myeloid Leukemia (AML): Different Treatment Strategies Versus a Common Standard Arm—Combined Prospective Analysis by the German AML Intergroup. *Journal of Clinical Oncology* 2012; **30**: 3604–3610.

2 Niederwieser D, Lang T, Krahl R, Heinicke T, Maschmeyer G, Al-Ali HK *et al.* Different treatment strategies versus a common standard arm (CSA) in patients with newly diagnosed AML over the age of 60 years: a randomized German inter-group study. *Ann Hematol* 2023; **102**: 547–561.

3 Stone RM, Mandrekar SJ, Sanford BL, Laumann K, Geyer S, Bloomfield CD *et al.* Midostaurin plus Chemotherapy for Acute Myeloid Leukemia with a *FLT3* Mutation. *New England Journal of Medicine* 2017; **377**: 454–464.

4 Erba HP, Montesinos P, Kim H-J, Patkowska E, Vrhovac R, Žák P *et al.* Quizartinib plus chemotherapy in newly diagnosed patients with FLT3-internal-tandem-duplication-positive acute myeloid leukaemia (QuANTUM-First): a randomised, double-blind, placebo-controlled, phase 3 trial. *The Lancet* 2023; **401**: 1571–1583.

5 Grimm J, Jentzsch M, Bill M, Goldmann K, Schulz J, Niederwieser D *et al.* Prognostic impact of the ELN2017 risk classification in patients with AML receiving allogeneic transplantation. *Blood Adv* 2020; **4**: 3864–3874.

6 Grimm J, Bill M, Jentzsch M, Beinicke S, Häntschel J, Goldmann K *et al.* Clinical impact of clonal hematopoiesis in acute myeloid leukemia patients receiving allogeneic transplantation. *Bone Marrow Transplant* 2019; **54**: 1189–1197.

7 Döhner H, Wei AH, Appelbaum FR, Craddock C, DiNardo CD, Dombret H *et al.* Diagnosis and management of AML in adults: 2022 recommendations from an international expert panel on behalf of the ELN. *Blood* 2022; **140**: 1345–1377.

8 Jentzsch M, Bill M, Grimm J, Schulz J, Goldmann K, Beinicke S *et al.* High *BAALC* copy numbers in peripheral blood prior to allogeneic transplantation predict early relapse in acute myeloid leukemia patients. *Oncotarget* 2017; **8**: 87944–87954.

9 Bill M, Grimm J, Jentzsch M, Kloss L, Goldmann K, Schulz J *et al.* Digital droplet PCR-based absolute quantification of pre-transplant NPM1 mutation burden predicts relapse in acute myeloid leukemia patients. *Ann Hematol* 2018; **97**: 1757–1765.

10 Lange T, Hubmann M, Burkhardt R, Franke G-N, Cross M, Scholz M *et al.* Monitoring of WT1 expression in PB and CD34+ donor chimerism of BM predicts early relapse in AML and MDS patients after hematopoietic cell transplantation with reduced-intensity conditioning. *Leukemia* 2011; **25**: 498–505.

11 Jentzsch M, Bill M, Grimm J, Schulz J, Beinicke S, Häntschel J *et al.* Prognostic Impact of Blood MN1 Copy Numbers before Allogeneic Stem Cell Transplantation in Patients with Acute Myeloid Leukemia. *Hemasphere* 2019; **3**. doi:10.1097/HS9.0000000000000167.

12 Backhaus D, Jentzsch M, Bischof L, Brauer D, Wilhelm C, Schulz J *et al.* Risk Stratification, Measurable Residual Disease, and Outcomes of AML Patients with a Trisomy 8 Undergoing Allogeneic Hematopoietic Stem Cell Transplantation. *Cancers (Basel)* 2021; **13**: 5679.

13 Glucksberg H, Storb R, Fefer A, Buckner CD, Neiman PE, Clift RA *et al.* Clinical manifestations of graft-versus-host disease in human recipients of marrow from HL-A-matched sibling donors. *Transplantation* 1974; **18**: 295–304.

14 Gray RJ. A Class of K-Sample Tests for Comparing the Cumulative Incidence of a Competing Risk. *The Annals of Statistics* 1988; **16**: 1141–1154.

15 R Core Team. R Core Team 2021 R: A language and environment for statistical computing. R foundation for statistical computing. . *R Foundation for Statistical Computing* 2022; **2**.

**Supplementary Tables**

**Supplementary Table S1.** Patient and HSCT characteristics

|  | **all**  **patients, n=144** | **Cyclophosphamide-TBI,**  **n=90** | **TBI-Cyclophosphamide,**  **n=54** | ***P*** |
| --- | --- | --- | --- | --- |
| **Age at HSCT**  median (age) | 43 (18-58) | 43 (18 – 56) | 42 (20 – 58) | 0.70 |
| **Sex, n (%)**  male  female | 80 (56)  64 (44) | 49 (54)  41 (46) | 31 (57)  23 (43) | 0.86 |
| **AML origin, n (%)**  secondary/tAML  *de novo* | 27 (19)  117 (81) | 17 (19)  73 (81) | 10 (19)  44 (82) | 1 |
| **ELN2022 risk, n (%)**  favorable  intermediate  adverse | 34 (34)  31 (31)  36 (36) | 20 (29)  24 (35)  25 (36) | 14 (44)  7 (22)  11 (34) | 0.27 |
| **Complex karyotype, n (%)**  absent  present | 111 (77)  18 (13) | 65 (80)  16 (20) | 46 (96)  2 (4) | 0.02 |
| ***NPM1* status, n (%)**  Wild type  mutated | 81 (72)  31 (28) | 50 (70)  21 (30) | 31 (76)  10 (24) | 0.66 |
| ***FLT3*-ITD, n (%)**  absent  present | 81 (70)  35 (30) | 49 (66)  25 (34) | 32 (76)  10 (24) | 0.30 |
| ***FLT3*-TKD, n (%)**  absent  present | 86 (82)  19 (18) | 59 (89)  7 (11) | 27 (69)  12 (31) | 0.02 |
| **HCT-CI, n (%)**  0  1-2  ≥ 3 | 74 (51)  35 (24)  34 (24) | 45 (50)  23 (26)  21 (24) | 29 (54)  12 (22)  13 (24) | 0.90 |
| **Remission at HSCT, n (%)**  1st CRc  later CRc  no CRc | 114 (79)  26 (18)  4 (3) | 68 (76)  19 (21)  3 (3) | 46 (85)  7 (13)  1 (2) | 0.48 |
| **MRD status at HSCT, n (%)**  MRD negative  MRD positive | 52 (71)  21 (29) | 32 (70)  14 (30) | 20 (74)  7 (26) | 0.79 |
| **Donor, n (%)**  matched related  matched unrelated  mismatched unrelated | 55 (38)  77 (53)  11 (8) | 32 (36)  51 (57)  7 (8) | 23 (43)  26 (49)  4 (8) | 0.64 |
| **Donor sex, n (%)**  female into male  all others | 19 (14)  120 (86) | 13 (15)  76 (85) | 6 (12)  44 (88) | 0.80 |
| **CMV serostatus, n (%)**  patient + / donor –  all others | 47 (33)  95 (66) | 29 (32)  61 (68) | 18 (35)  34 (65) | 0.85 |
| **Acute GvHD ≥ grade 2, n (%)**  absent  present | 98 (78)  27 (22) | 65 (80)  16 (20) | 33 (75)  11 (25) | 0.50 |
| **Chronic GvHD, n (%)**  absent  limited  extensive | 53 (56)  9 (9)  33 (35) | 30 (55)  3 (5)  22 (40) | 23 (58)  6 (15)  11 (28) | 0.19 |

*Abbreviations: AML, acute myeloid leukemia; CMV: cytomegaly virus; Cyclophosphamide-TBI: cyclophosphamide followed by total body irradiation; ELN: European Leukemia Net; GvHD: graft-versus-host disease; HCT-CI: hematopoietic cell transplantation-specific comorbidity index; HSCT: hematopoietic stem cell transplantation; MRD: measurable residual disease; TBI-Cyclophosphamide: total body irradiation followed by cyclophosphamide*

**Supplementary Table S2.** Multivariate analysis

|  | Cumulative incidence of relapse | | Non-relapse mortality | | Overall survival | |
| --- | --- | --- | --- | --- | --- | --- |
|  | **HR* (95% CI)** | ***P*** | **HR* (95% CI)** | ***P*** | **OR** (95% CI)** | ***P*** |
| Patient Sex  (female *vs* male) | - | - | 3.07 (1.10 – 8.59) | .03 | - | - |
| Disease origin  (*de novo vs* sAML/tAML) | - | - | 0.34 (0.13 – 0.91) | .03 | - | - |
| ELN2022 genetic risk  (adverse *vs* intermediate *vs* favorable) | 2.44 (1.66 - 3.56) | <.001 | - | - | 2.09 (1.29 – 3.38) | .003 |
| Age at HSCT  (> 40 years *vs* 40 years) | - | - | 5.46 (1.23 – 24.26) | .03 | 2.95 (1.30 – 6.70) | .01 |
| Remission status at HSCT  (no CRp *vs* CRp) | 10.69 (4.20 – 27.18) | <.001 | - | - | 7.78 (2.01 – 30.05) | .003 |

*Abbreviations: AML, acute myeloid leukemia; CI, confidence interval; CRp, composite complete remission; ELN2022, European LeukemiaNet 2022 risk; HSCT, hematopoietic stem cell transplantation; sAML, secondary AML; tAML, treatment-related AML .*

*HR, hazard ratio, <1 (>1) indicate lower (higher) risk of relapse for the first category listed for the dichotomous variables.
**OR, odds ratio, <1 (>1) indicate lower (higher) chance of survival for the first category listed for the dichotomous variables.
Variables considered in the models were those with available data for at least 50% of patients that were significant at α=0.10 in univariate analyses.
For CIR endpoint, variables considered were: ELN2022 risk group (adverse *vs* intermediate vs favorable), remission status at HSCT (no CRp *vs* CRp), and CMV risk (patient positive/donor negative *vs* all others). For NRM endpoint, variables considered were: patient sex (female *vs* male), disease origin (de novo *vs* sAML/tAML), and age at HSCT (> 40 years *vs* 40 years). For OS endpoint, variables considered were: patient sex (female *vs* male), disease origin (de novo *vs* sAML/tAML), ELN2022 risk group (adverse *vs* intermediate vs favorable), remission status at HSCT (no CRp *vs* CRp), age at HSCT (> 40 years *vs* 40 years), and CMV risk (patient positive/donor negative *vs* all others).

**Supplementary Figures**

**Supplementary Figure S1**


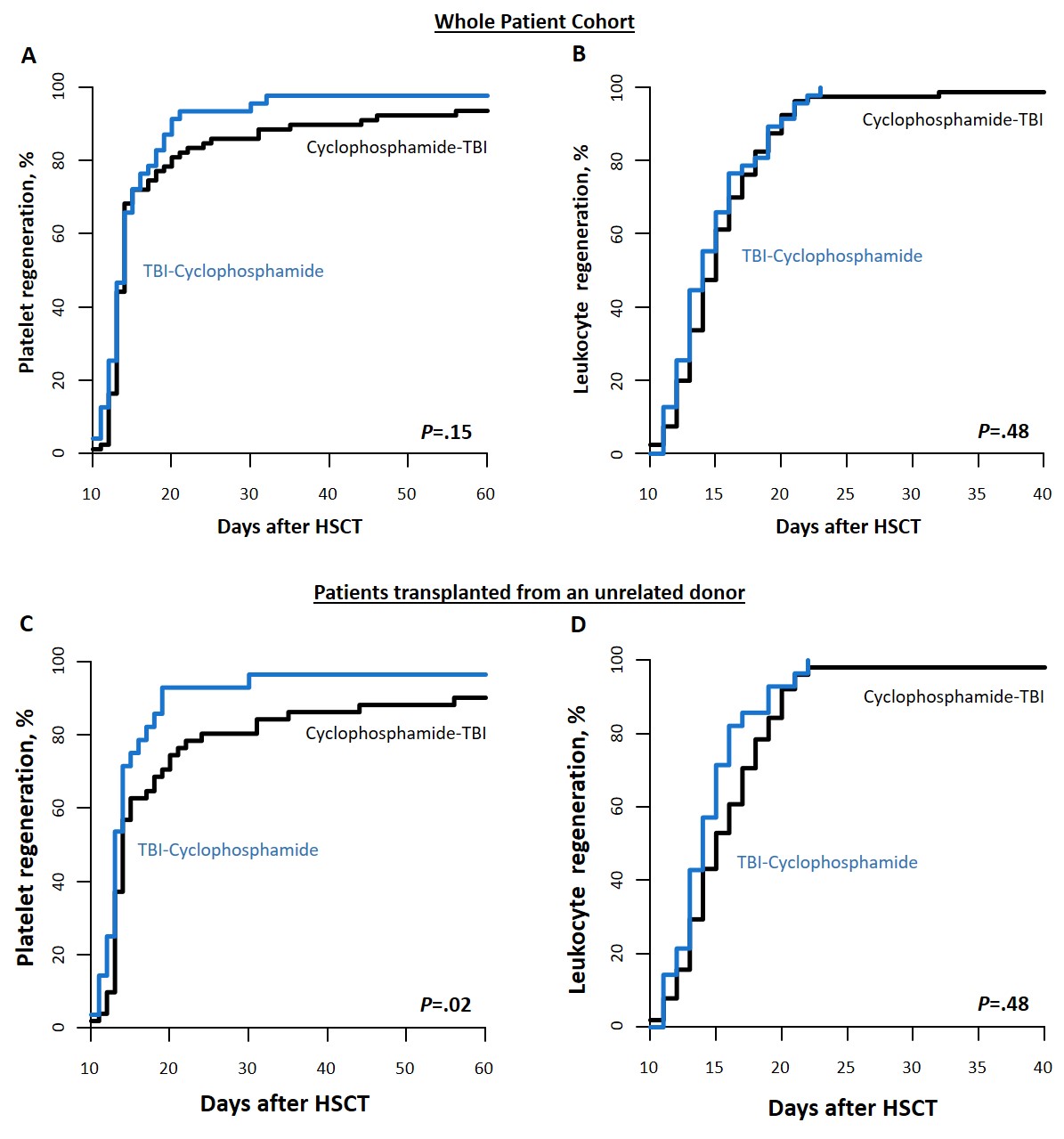


**Supplementary Figure S1. Time to engraftment according to the sequence of cyclophosphamide and total body irradiation. (A)** Time to platelet engraftment and **(B)** time to leukocyte engraftment in the whole patient cohort and **(C)** Time to platelet engraftment and **(D)** time to leukocyte engraftment in patients transplanted from an unrelated donor.

**Supplementary Figure S2**

**Supplementary Figure S2. Time to engraftment according to the sequence of cyclophosphamide and total body irradiation in patients transplanted from a matched sibling donor. (A)** Time to platelet engraftment and **(B)** time to leukocyte engraftment.

**Supplementary Figure S3**


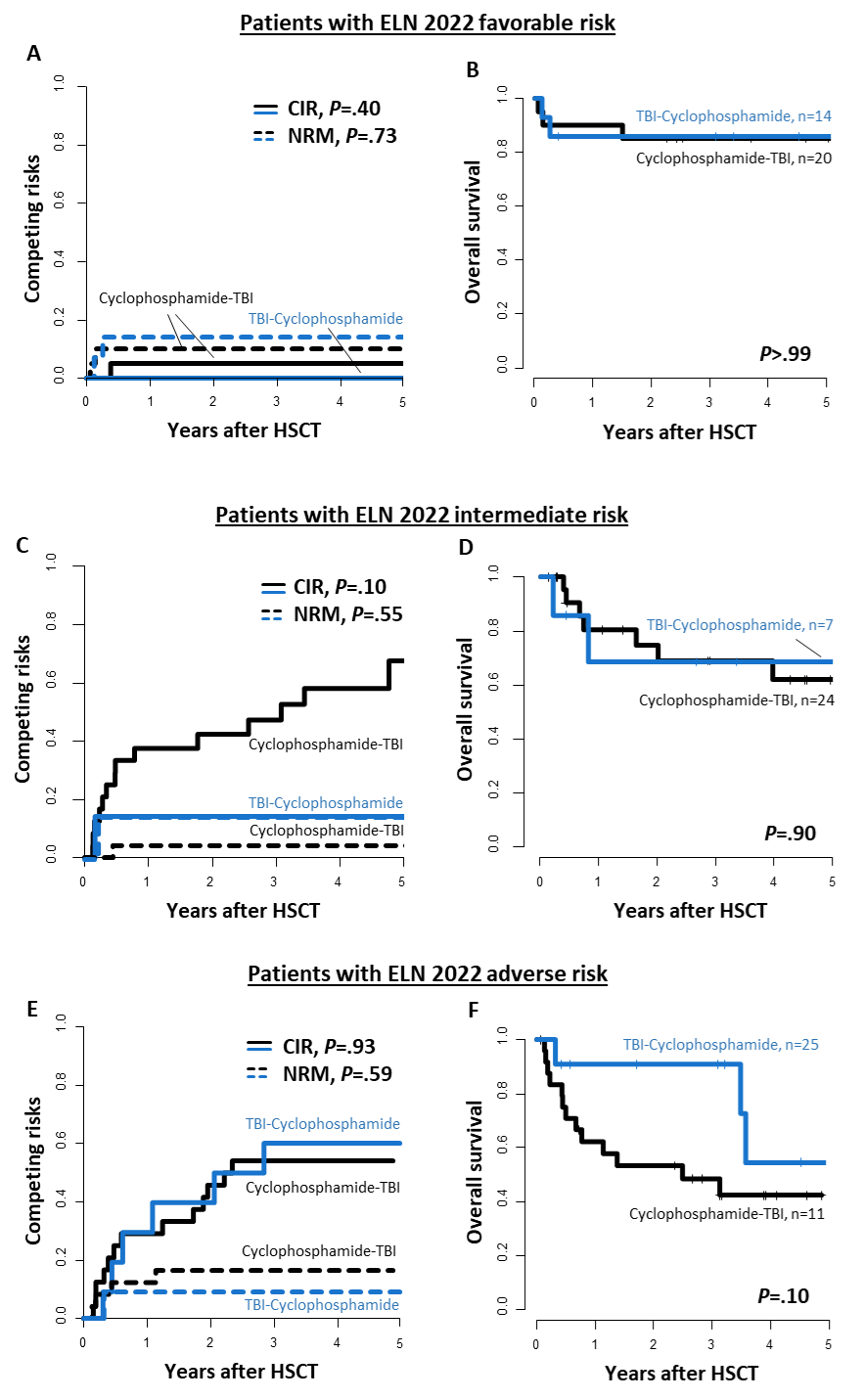


**Supplementary Figure S3. Cumulative incidence of relapse (CIR), non-relapse mortality (NRM) and overall survival (OS) according to the sequence of cyclophosphamide and total body irradiation for the distinct ELN 2022 risk groups. (A)** Competing risks, and **(B)** OS in patients with favorable ELN 2022 risk, **(C)** Competing risks and **(D)** OS in patients with intermediate ELN 2022 risk, and **(E)** Competing risks and **(F)** OS and in patients with adverse ELN 2022 risk.

**Supplementary Figure S4**


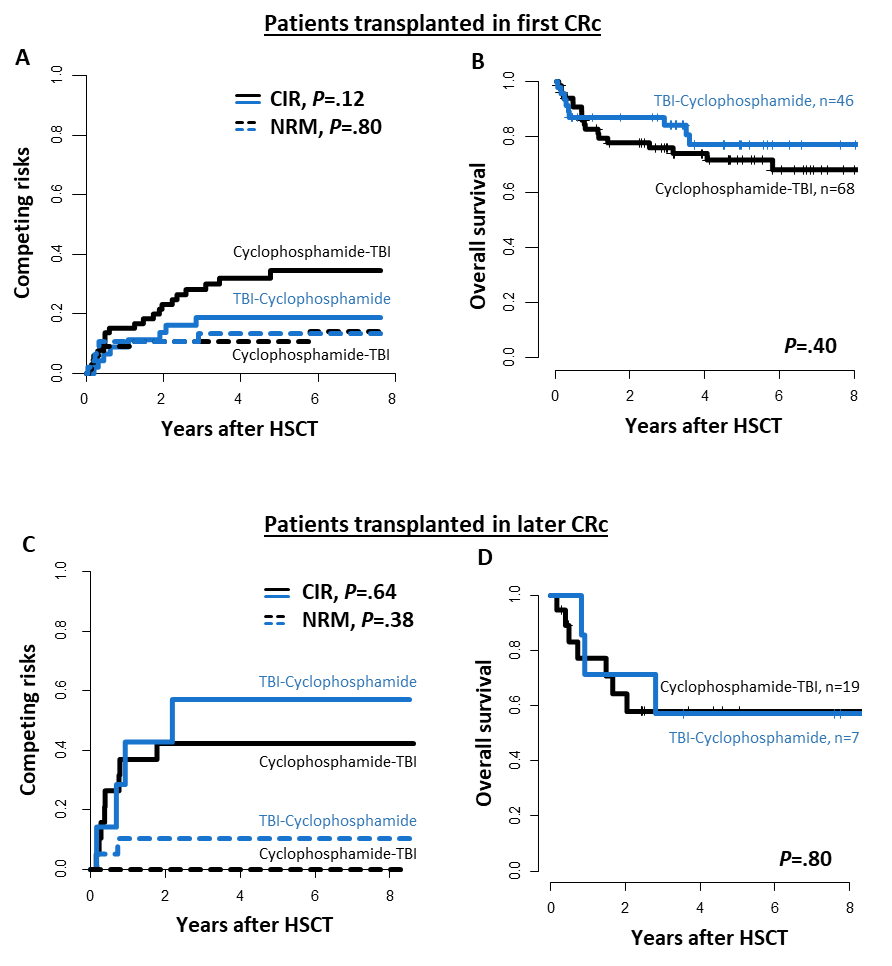


**Supplementary Figure S4. Cumulative incidence of relapse (CIR), non-relapse mortality (NRM) and overall survival (OS) according to the sequence of cyclophosphamide and total body irradiation in patients in first or second remission at HSCT.** **(A)** Competing risks, and **(B)** OS in patients transplanted in first CRc, and **(C)** Competing risks and **(D)** OS in patients transplanted in second CRc.

**Supplementary Figure S5**

**
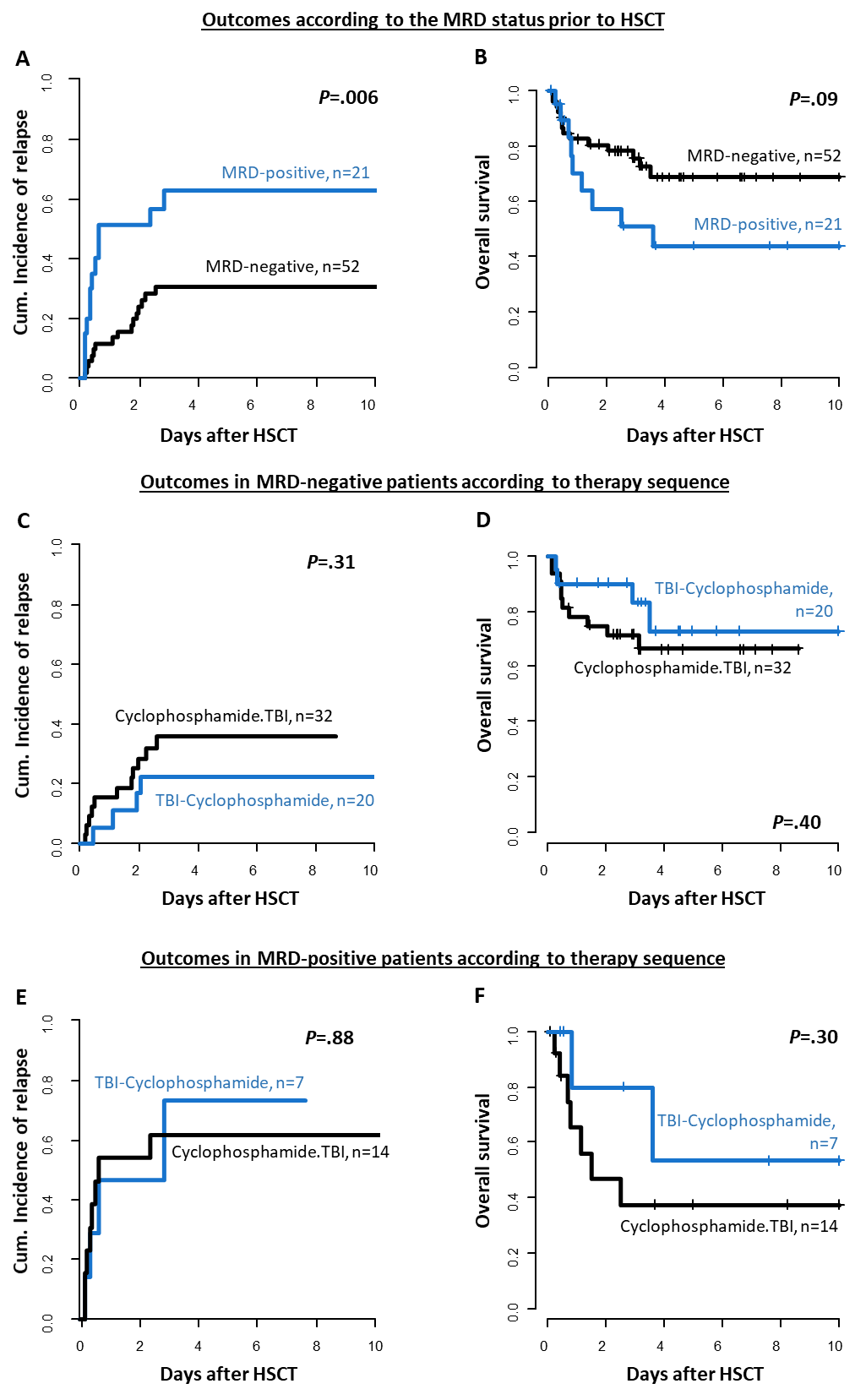
**

**Supplementary Figure S5. Outcomes according to the sequence of cyclophosphamide and total body irradiation as well as the MRD status at HSCT. (A)** Cumulative incidence of relapse (CIR), and **(B)** Overall survival (OS) in all patients according to the pre-HSCT MRD status. **(C)** CIR and **(D)** OS according to the sequence of cyclophosphamide and total body irradiation in MRD-negative patients and **(C)** CIR and **(D)** OS according to the sequence of cyclophosphamide and total body irradiation in MRD-positive patients.

**Supplementary Figure S6**


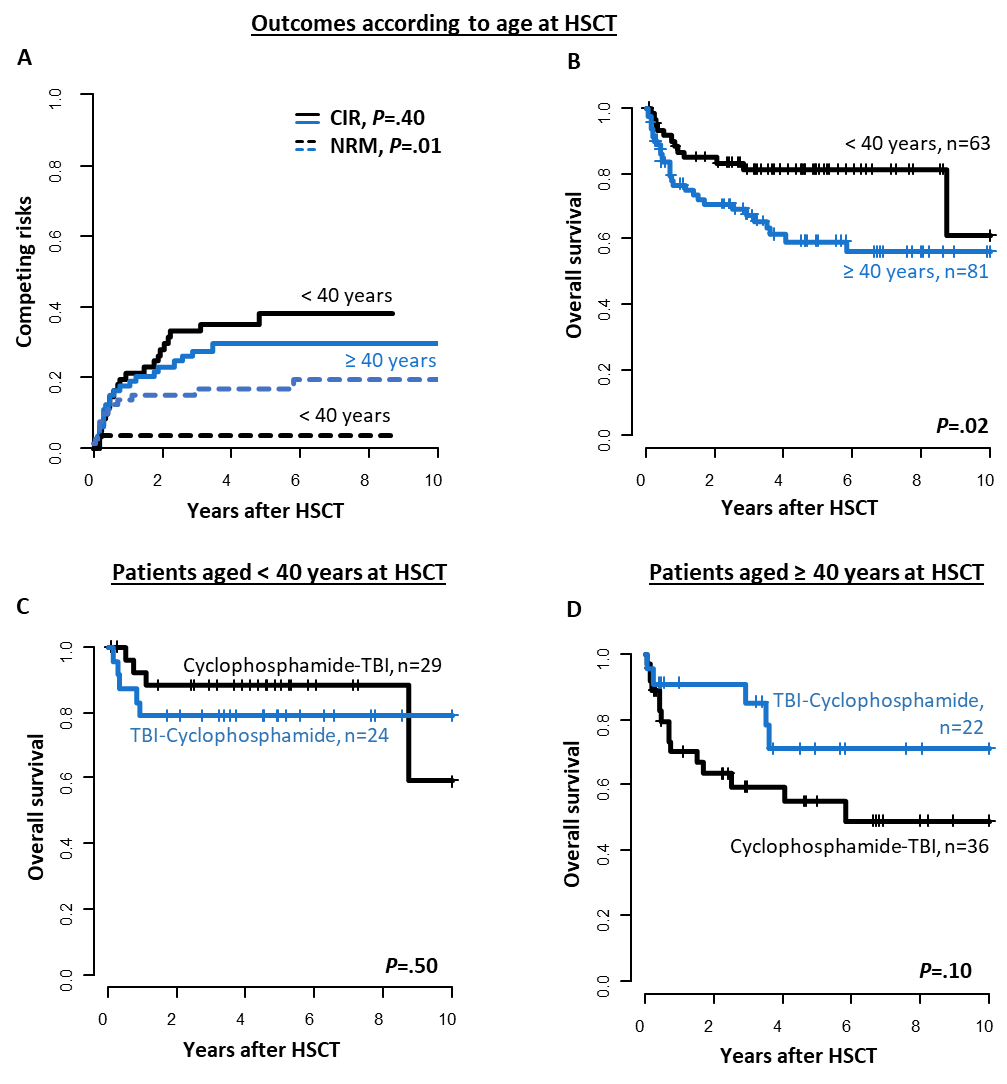


**Supplementary Figure S6. Outcomes according to the sequence of cyclophosphamide and total body irradiation as well as age at HSCT. (A)** Cumulative incidence of relapse (CIR), non-relapse mortality (NRM), and **(B)** Overall survival (OS) in all patients according to the age at HSCT. **(C)** OS in patients younger and **(D)** OS in patients older than 40 years not harboring a complex karyotype according to the sequence of cyclophosphamide and total body irradiation.
